# Idiosyncratic preferences in transparent motion and binocular rivalry are dissociable

**DOI:** 10.1167/jov.20.12.3

**Published:** 2020-11-06

**Authors:** Byung-Woo Hwang, Alexander C. Schütz

**Affiliations:** Allgemeine und Biologische Psychologie, Philipps-Universität Marburg, Marburg, Germany; Center for Mind, Brain and Behavior, Philipps-Universität Marburg, Marburg, Germany

**Keywords:** binocular rivalry, bistable perception, individual differences, transparent motion

## Abstract

Previous studies revealed that there are idiosyncratic preferences to perceive certain motion directions in front during motion transparency depth rivalry (Mamassian & Wallace, 2010; Schütz, 2014). Meanwhile, other studies reported idiosyncratic preferences in binocular rivalry during the onset stage (Carter & Cavanagh, 2007; Stanley, Carter, & Forte, 2011). Here we investigated the relationship of idiosyncratic preferences in transparent motion and binocular rivalry. We presented two dot clouds that were moving in opposite directions. In the transparent motion condition, both dot clouds were presented to both eyes and participants had to report the dot cloud they perceived in front. In the binocular rivalry condition, the dot clouds were presented to different eyes and participants had to report the dominant dot cloud. There were strong idiosyncratic directional preferences in transparent motion and rather weak directional preferences in binocular rivalry. In general, binocular rivalry was dominated by biases in contrast polarity, whereas transparent motion was dominated by biases in motion direction. A circular correlation analysis showed no correlation between directional preferences in transparent motion and binocular rivalry. These findings show that idiosyncratic preferences in a visual feature can be dissociated at different stages of processing.

## Introduction

Perception includes the interpretation of ambiguous or conflicting sensory information. Many ambiguous visual stimuli have been discovered in visual perception studies (for a review, see Brascamp, Sterzer, Blake, & Knapen, 2018). Famous examples are binocular rivalry, in which different visual stimuli are shown to the two eyes (Blake, 2001; Wheatstone, 1838), or the Necker cube, in which a two-dimensional (2D) visual stimulus could be perceived in two different three-dimensional (3D) orientations (Necker, 1832; Long & Toppino, 2004). Transparent motion is another example of an ambiguous stimulus. When there are two dot clouds moving in different directions at the same space, humans perceive two transparent surfaces of coherently moving dots sliding over each other (Andersen, 1989). Because there is no actual depth cue about which of the two surfaces is in front, the subjective interpretation could vary arbitrarily. This kind of bistable depth ordering in transparent motion is called “motion transparency depth rivalry” (Chopin & Mamassian, 2011).

Perceptual biases are a widespread phenomenon in visual perception (for reviews see Klink, van Wezel, & van Ee, 2012; Scocchia, Valsecchi, & Triesch, 2014) and can be a useful research tool to investigate the processing of information in the visual system. Perceptual biases can reveal assumptions of the visual system about the most likely state of the world, as for instance in the light-from-above prior (Mamassian & Goutcher, 2001) or the valuation of different interpretations, for instance when rivalry stimuli are coupled with different rewards (Wilbertz, van Slooten, & Sterzer, 2014; Marx & Einhäuser, 2015). Perceptual biases become even more interesting when they are not shared across the population but show large differences between individuals. Recent studies revealed that there are idiosyncratic depth order preferences to perceive certain directions in front during motion transparency depth rivalry (Mamassian & Wallace, 2010; Schütz, 2014; Wexler, Duyck, & Mamassian, 2015; Goutcher, 2016). Interestingly, these individual preferences exhibit long-term stability. Idiosyncratic perceptual biases have not only been reported for transparent motion. Recent binocular rivalry studies reported idiosyncratic preferences in binocular rivalry during the onset stage (Carter & Cavanagh, 2007; Stanley, Carter, & Forte, 2011). Using brief presentations, Carter and Cavanagh (2007) showed strong onset biases within any given visual location while presenting stimuli with different orientations and colors to the two eyes. Also, these biases were highly idiosyncratic across participants but stable over several weeks.

A few studies investigated whether individual preferences are shared among different tasks or whether they are independent of one another (Schütz, 2014; Wexler, Duyck, & Mamassian, 2015; Brascamp, Becker, & Hambrick, 2018; Cao, Wang, Sun, Engel, & He, 2018). Here we set out to compare individual preferences in transparent motion and binocular rivalry. This is interesting because of three reasons. First, although the resulting percept is very different in both cases, the perceptual biases might be related in both cases to the strength of motion representation, and therefore might share the same neural basis. Previous studies suggested that both the perception of transparent motion and of binocular rivalry might involve neural competition at similar levels of the visual pathway, for example the primary visual cortex (V1) (Andersen & Bradley, 1998; Tong, Meng, & Blake, 2006). Furthermore, several other studies observed perceptual and behavioral dominance of one of the two motion surfaces in transparent motion (Lankheet & Verstraten, 1995; Mestre & Masson, 1997; Valdes-Sosa, Cobo, & Pinilla, 2000): for example, human observers show larger motion after effects of the attended motion surface compared with the unattended motion surface (Lankheet & Verstraten, 1995); there is a correlation between the attended motion surface and the direction of the slow-phase in optokinetic nystagmus (Mestre & Masson, 1997); and it is difficult to rapidly shift attention from one motion surface to the other (Valdes-Sosa, Cobo, & Pinilla, 2000). These studies suggest that one of the two surfaces in transparent motion is perceptually dominant and that perceptual biases in transparent motion might be related to biases in perceptual dominance in binocular rivalry. Second, several recent studies (Brascamp, Becker, & Hambrick, 2018; Cao, Wang, Sun, Engel, & He, 2018; Steinwurzel, Animali, Cicchini, Morrone, & Binda, 2020) compared the temporal aspects of multistability in binocular rivalry and structure-from-motion (Wallach & O'Connell, 1953), which is similar to transparent motion. Especially because these studies showed inconsistent evidence about the correlation in percept durations between binocular rivalry and structure-from-motion, we believe it is interesting to compare the two classes of stimuli also with respect to their spatial aspects, that is, their directional biases. Third, a previous study (Schütz & Mamassian, 2016) showed that directional biases in transparent motion depend on one-dimensional (1D) rather than 2D motion signals. This suggests a neural origin either in the responses of neurons in V1 or in the early responses of neurons in the middle temporal area (MT) because the aperture problem (Wallach, 1935) is solved only afterward (Pack & Born, 2001; Pack, Livingstone, Duffy, & Born, 2003). Because eye rivalry rarely occurs beyond V1 (Blake, 1989; Baker & Graf, 2009; Klink, Brascamp, Blake, & van Wezel, 2010; Brascamp, Sohn, Lee, & Blake, 2013), comparing directional preferences in binocular rivalry and transparent motion might provide additional constraints on the neural origin of directional preferences. If transparent motion and binocular rivalry show similar directional biases in each individual, this would suggest that the idiosyncratic bias in transparent motion originates from V1. Otherwise, if the two stimuli show dissimilar bias patterns, then the early responses of neurons in MT might be responsible for the directional bias in transparent motion. Consequently, our study would provide new insights about the spatial relationship between transparent motion and binocular rivalry and allow us to further narrow down the neural origin of perceptual biases in the two phenomena.

## Methods

### Experiment 1: Biases without calibration

#### Participants

We recorded 13 participants (aged between 18 and 26 years, 12 women) for this experiment. All of them had normal or corrected to normal vision and gave prior informed consent. Participants were paid for participation (8€/h) or given course credit. All experiments were conducted in accordance with the ethical standards laid down in the 1964 Declaration of Helsinki and were approved by the local ethics committee (proposal number 2015-35k).

#### Equipment

Experiments were conducted using the Psychtoolbox (Brainard, 1997; Pelli, 1997) in MATLAB (The MathWorks, Natick, MA) and presented on a VIEWPixx monitor (VPixx Technologies Inc., Saint-Bruno, Quebec, Canada) at a viewing distance of 68.5 cm. The monitor had a spatial resolution of 1920 × 1080 pixels and a size of 51.5 × 29 cm. We recorded eye movements of both eyes using a desktop mounted EyeLink 1000 (SR Research Ltd., Ontario, Canada) with a sampling rate of 1000 Hz and the Eyelink Toolbox (Cornelissen, Peters, & Palmer, 2002). A mirror stereoscope (Wheatstone, 1838) consisting of four first surface mirrors with a diameter of 50.8 mm (Thorlabs Inc. Newton, NJ) was used to bring the views of the two eyes into alignment. The eye tracker was recording the eyes directly beneath the mirrors of the stereoscope.

#### Visual stimuli

Random-dot kinematograms (RDKs) were composed of black and white dots (0.15 * 0.15 degrees of visual angle [dva]) for each motion dot cloud layer on a gray background. The dots moved at a speed of 10 dva/s and had a limited lifetime of 200 ms. The initial lifetime was randomized for each dot separately. In the transparent motion condition, each RDK was composed of two spatially overlapping motion dot clouds, moving in opposite directions. In the binocular rivalry condition, the composition of the two motion dot clouds was similar as in the transparent motion condition, but the two motion dot clouds were presented to different eyes. Each RDK was presented for 400 ms. The dot density of the RDK was one dot/dva^2^ in the transparent motion condition and five dot/dva^2^ in the binocular rivalry condition. The dot density was higher in the binocular rivalry condition to optimize rivalry and to achieve a clear and stable dominance. Motion was displayed within stationary, circular apertures with a radius of 5 dva. The aperture was surrounded by a 1.5 dva thick noise pattern to facilitate the appearance of an aperture and the alignment of two eyes. A red crosshair was presented as an fixation target throughout the trial (Thaler, Schütz, Goodale, & Gegenfurtner, 2013).

#### Design

The motion direction of the dot clouds was varied in 24 steps of 15° from 0° to 345°. In the transparent motion condition, each condition was repeated 10 times, leading to a total of 240 trials. In the binocular rivalry condition, we presented two different polarities (black and white dot clouds) for each eye, and each condition was repeated five times, leading to a total of 240 trials.

#### Experimental procedure

Participants started each trial by pressing the space bar, which triggered the presentation of two motion dot clouds that were moving in opposite directions. Participants performed two consecutive experimental conditions. In the transparent motion condition, both dot clouds were presented to both eyes and participants had to report the color of the dot cloud they perceived in front. In the binocular rivalry condition, the two dot clouds were presented to different eyes and participants had to report the color of the dominant dot cloud. The sequence of the two conditions was counterbalanced across participants.

### Experiment 2: Eliminated polarity bias

To remove potential biases for contrast polarity, we used the same contrast polarity for both dot clouds in Experiment 2.

#### Participants

We recorded 13 participants (aged between 19 and 31 years, 11 women) for this experiment.

#### Visual stimuli

Stimuli were identical to Experiment 1, with the following changes: RDKs were composed of only black dots (0.15 * 0.15 dva) for each motion dot cloud layer on a gray background. Each RDK was composed of two layers, moving in opposite directions and presented for 600 ms.

#### Design

The motion direction of the dot clouds was varied in 24 steps of 15° from 0° to 345°, and each condition was repeated 10 times, leading to a total of 240 trials for each condition.

#### Experimental procedure

In the transparent motion condition, participants had to report the motion direction of the dot cloud they perceived in front. In the binocular rivalry condition, participants had to report the motion direction of the dominant dot cloud. After each trial, a line extending from the center of the display to the outer edge of the stimulus aperture, was pointing in one of the two motion directions present in that trial. Participants could toggle the line between the two motion directions with one button and confirm their selection with another button.

### Experiment 3: Calibrated polarity bias

In Experiment 3, to facilitate perceptual dominance in the binocular rivalry condition, we used two different contrast polarities for the two dot clouds as in Experiment 1. To minimize potential biases for contrast polarity, we calibrated the relative contrast of white and black dots relative to the gray background for each participant.

#### Participants

We recorded 13 participants (aged between 19 and 32 years, 11 women) for this experiment.

#### Visual stimuli

Stimuli were identical to Experiment 1, with the following changes: RDKs were composed of two dot clouds of black and white dots (0.22 * 0.22 dva), respectively. Each RDK was composed of two layers, moving in opposite directions and presented for 600 ms. The dot density of the RDK was one dot/dva^2^ in transparent motion condition, and the dot density was six dot/dva^2^ in binocular rivalry condition. To achieve a clear and stable dominance, we used a higher dot density in the binocular rivalry condition.

#### Design

The motion direction of the dot clouds was varied in 24 steps of 15° from 0° to 345°. In the transparent motion condition, each condition was repeated 10 times, leading to a total of 240 trials. In the binocular rivalry condition, we presented two different polarities (black and white dot clouds) for each eye, and each condition was repeated five times, leading to a total of 240 trials. Before the main experiment, we additionally performed a calibration session (as explained in “*Calibration session*”).

#### Experimental procedure

The experimental procedure of the main experiment was the same as in Experiment 2.

#### Calibration session

Before the main experiment, observers participated in a calibration session. The goal of this session was to identify for each participant individually the contrast of white and black relative to the gray background where white and black dot clouds are equally often perceived in front/dominant. We used interleaved staircases to find these contrast values. The contrast started from 100% for one of the two polarities (e.g., full white) and 10% contrast for the other polarity (e.g., gray slightly darker than the background). When the participant perceived one of the dot clouds in front in transparent motion (or dominant in binocular rivalry), we decremented the contrast for the selected polarity and incremented the contrast for the unselected polarity by 10% in the next trial (e.g., from 100% white and 10% black to 90% white and 20% black). The maximum contrast level of one polarity was 90% and the contrast of both polarities was coupled such that the mean of both contrasts remained constant at 55%. In one of two interleaved staircases, the calibration started at 100% white and 10% black and in the other staircase, the calibration started from at 10% white and 100% black. The motion direction of the dot clouds was varied in eight steps of 45° from 0° to 315°. In the transparent motion condition, each condition (two staircases × eight different motion directions) was repeated three times, leading to a total of 48 trials for the calibration session. After the calibration session, we measured the mean contrast value in the last eight trials (which included eight different motion directions) for each staircase. Then, for each polarity, we measured the mean contrast from the two staircases. In binocular rivalry, additionally, we needed to calibrate two sets of contrast values for each eye, and each condition (two eye-of-origin × two staircases × eight different motion directions) was repeated three times, leading to a total of 96 trials for the calibration session. After the calibration session, we measured the mean contrast similar as in the transparent motion condition, additionally considering the eye-of-origin condition. The experimental procedure of the calibration session was similar as in Experiment 2.

### Experiment 4: Calibrated polarity bias and eliminated eye-of-origin bias

In Experiment 4, to minimize potential biases for eye-of-origin, we spilt each of the two dot clouds into two parts and showed them to different eyes. In addition, we performed the polarity contrast calibration as in Experiment 3 to minimize potential effects of contrast polarity.

#### Participants

We recorded 13 participants (aged between 19 and 32 years, 12 women) for this experiment.

#### Visual stimuli

RDKs were composed of black and white dots (0.22 * 0.22 dva) for each motion dot cloud layer on a gray background. In transparent motion, RDKs were presented for 600 ms, and in binocular rivalry for 900 ms. The dot density of the RDK was one dot/dva^2^ in transparent motion and six dot/dva^2^ in binocular rivalry. To achieve a clear and stable dominance, we used a longer presentation duration and a higher dot density in the binocular rivalry condition. Each dot cloud was split in half along the axis of motion direction in this trial. This means that the splitting axis varied across trials. The two halves of each dot cloud were presented to different eyes, such that each motion direction was present in both eyes, but not at the same location in space.

#### Design

The motion direction of the dot clouds was varied in 24 steps of 15° from 0° to 345°, and for both transparent motion and binocular rivalry conditions, each condition was repeated ten times, leading to a total of 240 trials. Before the main experiment, we additionally performed a calibration session (as explained in “*Calibration session*”).

#### Experimental procedure

The procedure of the main experiment was the same as in Experiment 2.

### Data analyses

#### Modeling

We calculated individual preferences for contrast polarity, eye-of-origin, and motion direction separately for each experimental condition. All biases were calculated in isolation while ignoring the other biases and then transformed to the same scale to make them comparable to each other. This corresponds conceptually to an analysis of the main effects of the biases.

The proportion of front choices in the transparent motion condition (or dominant choices in the binocular rivalry condition) was calculated as a function of motion direction. Proportion of choices were calculated as a function of motion direction and strength of preference was analyzed using a cosine model (Mamassian & Wallace, 2010; Schütz, 2014) (Equation 1). This model contains two free parameters: the preferred direction (θ_*m*_) and the magnitude of directional preferences (*b_dir_*). The preferred direction corresponds to the direction that is seen most often in front in the transparent motion condition and more dominant in the binocular rivalry condition. The magnitude of preferences was constrained within 0 to 20. The model was fit to each participant and each experimental condition separately.

(1) $$y_{i}=b_{dir}\cos\left(\theta -\theta_{m}\right),$$

The internal model responses (*y_i_*) were transformed into a proportion of choices (*y_e_*) using an inverse logit model (Equation 2):

(2) $$y_{e}=\frac{e^{y_{i}}}{1+e^{y_{i}}},$$

**Figure 1.**
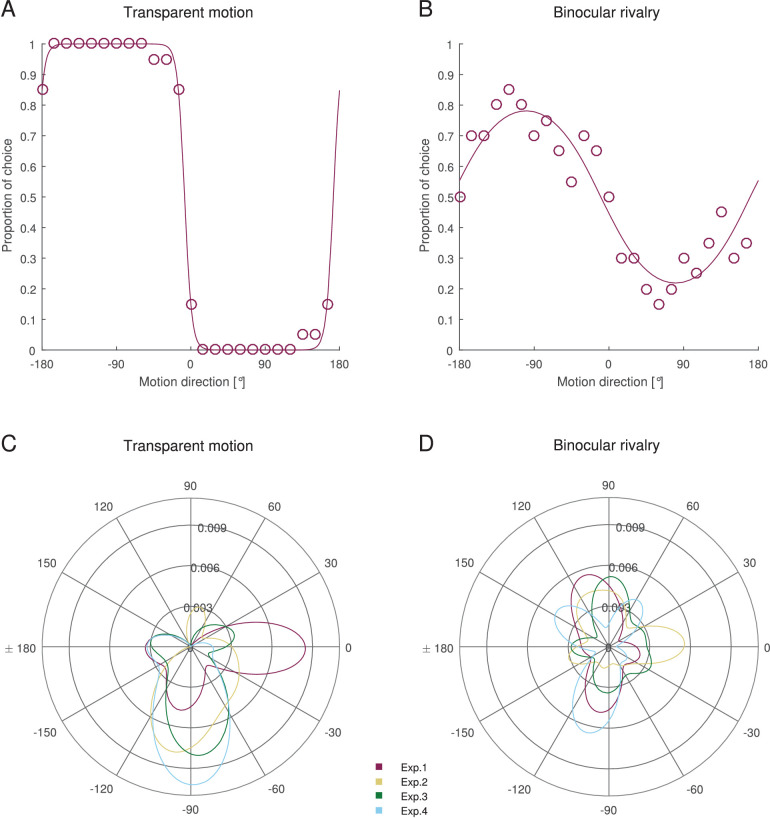
Individual direction bias and distribution of direction biases. (A and B) Individual data of observer #99 in Experiment 1. Each dot indicates the proportion of choices for one motion direction. Solid lines indicate the preferences from the fitted model. (A) Proportion of seen in front as a function of motion direction in the transparent motion condition. The observer had a preferred motion direction of –97.541 and a direction bias strength of 0.5. The observer had a polarity bias strength of 0.333. (B) Proportion of seen dominant as a function of motion direction in binocular rivalry condition. The observer had a preferred motion direction of –99.746 and a bias strength of 0.281. The observer had a polarity bias strength of 0.046 and an eye-of-origin bias strength of 0.279. (C and D) Distribution of preferred motion directions of all observers in all experiments. Histograms are smoothed using a 20°-wide kernel density estimation. The *r*-axis indicates estimated probability density. Colors indicate different experiments. (A and C) Transparent motion. (B and D) Binocular rivalry.

**Figure A1.**
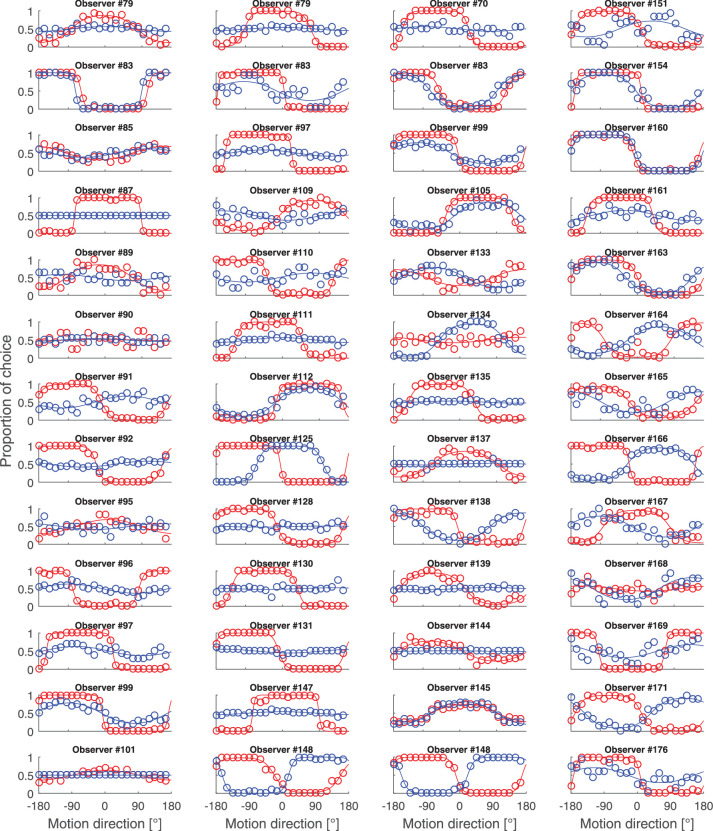
Individual participants' data. Proportion of seen in front as a function of motion direction in transparent motion condition (Red). Proportion of seen dominant as a function of motion direction in binocular rivalry condition (Blue). Each dot indicates the proportion of choice from behavioral data. Solid line indicates fitted model. Each column refers to Experiments 1 to 4. Please note that rows do not represent single observers because different observers participated in the experiments.

In Figures 1A and B, we show data and the fitted model of a representative individual participant in the transparent motion and binocular rivalry conditions. Data from all individual participants are shown in Figure A1.

**Figure 2.**
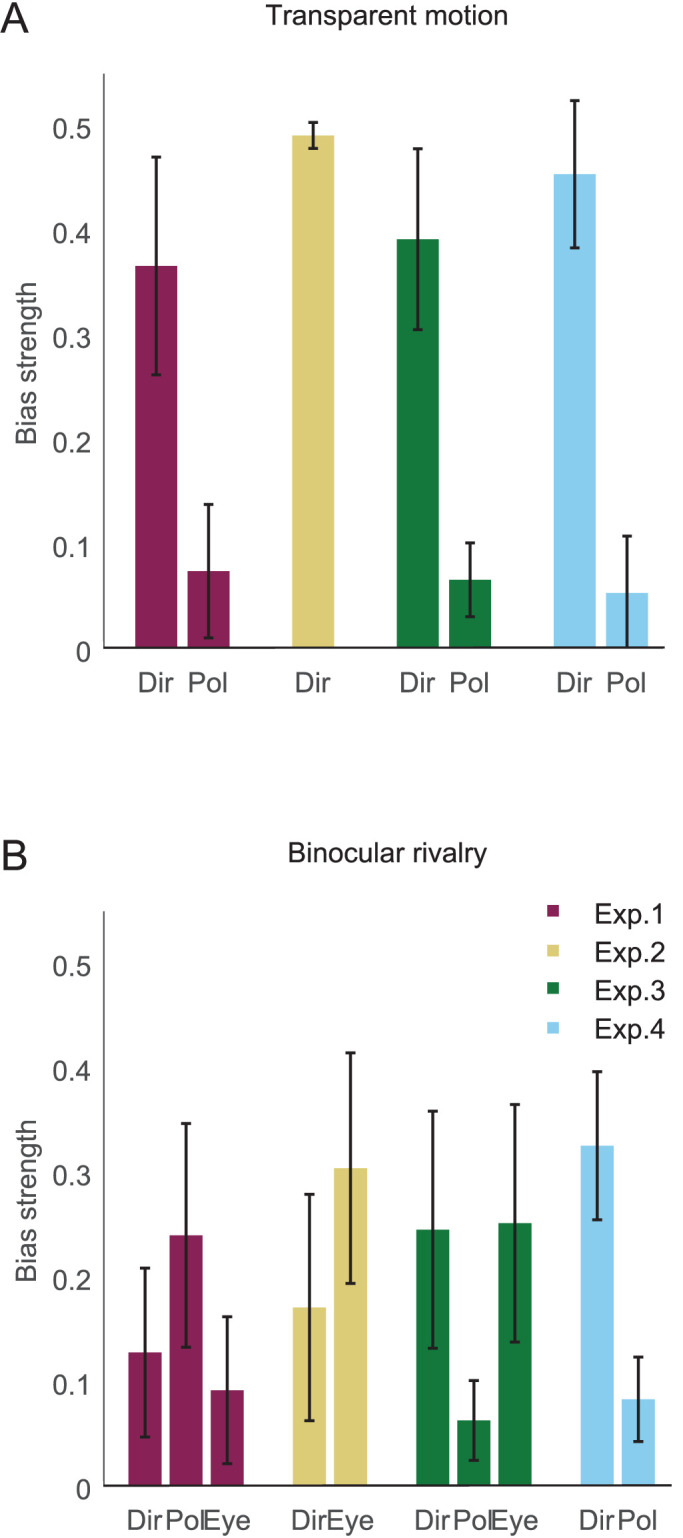
Absolute bias strength (A) in transparent motion condition and (B) in binocular rivalry condition. (Dir: direction bias; Pol: polarity bias; Eye: eye-of-origin bias). Error bars indicate 95% between-subjects confidence intervals.

For the polarity bias, we calculated the proportion of responses reporting the white motion dot cloud as seen in front in transparent motion condition (or dominant in binocular rivalry condition). Because this proportion scale is inconvenient to interpret, we subtracted 0.5 from all values, such that zero indicates completely balanced responses and the sign indicates the direction of the bias: negative values for biases toward black and positive values for biases toward white. In Figure 2, we show the absolute polarity bias values to compare the bias strength itself without considering the direction of the bias.

For the eye-of-origin bias, we calculated the proportion of responses reporting the stimulus on the right eye as seen dominant in binocular rivalry. Then we subtracted 0.5 from each value in the same way as for the polarity bias, such that negative values indicate biases toward the left eye and positive values indicate biases toward the right eye and zero completely balanced responses. In Figure 2, we show absolute eye-of-origin bias values to compare the bias strength itself without considering the direction of the bias.

To compare the strength of biases for motion direction, polarity, and eye-of-origin on the same scale, we transformed the magnitude of directional preferences (*b_dir_*; in Equation 1) using the inverse logit function (Equation 2), similar as described earlier. Then we subtracted 0.5 from each value of the transformed direction bias to set the same scale for direction bias values and the other bias values.

#### Correlation between preferred directions

To test for correlations between preferred directions we used a circular correlation coefficient analogous to a Pearson correlation coefficient (Jammalamadaka & Sengupta, 2001), with α and β as the two samples and μ_α_ and μ_β_ as their corresponding means (Equation 3). The circular statistics toolbox (Berens, 2009) was used to calculate this correlation coefficient.

(3) $$\;p_{c}\left(\propto ,\beta \right)=\;\frac{\sum \sin\left(\alpha -\mu_{\alpha }\right)\sin\left(\beta -\mu_{\beta }\right)}{\sqrt{\sum \left(\mathrm{si}n^{2}\left(\alpha -\mu_{\alpha }\right)\right)\sum \left(\mathrm{si}n^{2}\left(\beta -\mu_{\beta }\right)\right)}},\;$$

To remove the direction from the preferences and to restrict the correlation analysis to their axes, we normalized the preferred directions in binocular rivalry by adding 180° whenever the absolute difference between transparent motion and binocular rivalry preferences exceeded 90°. As a result, the two preferences of each observer point toward the same half circle, thereby preserving axes differences while removing direction differences. For instance, if an observer has a preference of 0° in transparent motion and 120° in binocular rivalry, the normalized preference in binocular rivalry would be –60°. The circular correlation coefficient with these normalized preferences cannot be tested against zero because the normalization itself imposes a certain degree of correlation. Instead, we generated an empirical distribution of correlation coefficients that does not contain a systematic relationship between the two preferences by randomly reassigning the preferences in transparent motion and binocular rivalry across observers in 1000 separate datasets. For each of those datasets, the normalization procedure was applied, and a circular correlation coefficient computed. The correlation coefficient of the actual dataset was then compared with the distribution of the correlation coefficients based on the reshuffled datasets.

## Results

### Experiment 1: Biases without calibration

Replicating previous findings (Mamassian & Wallace, 2010; Schütz, 2014; Wexler, Duyck, & Mamassian, 2015), the motion direction perceived in front was generally biased toward rightward or downward motion (Figure 1C), and participants showed strong direction biases (Figure 2A). In binocular rivalry, the preferred directions were clustered at cardinal axes but still spread across all directions (Figure 1D).

**Figure 3.**
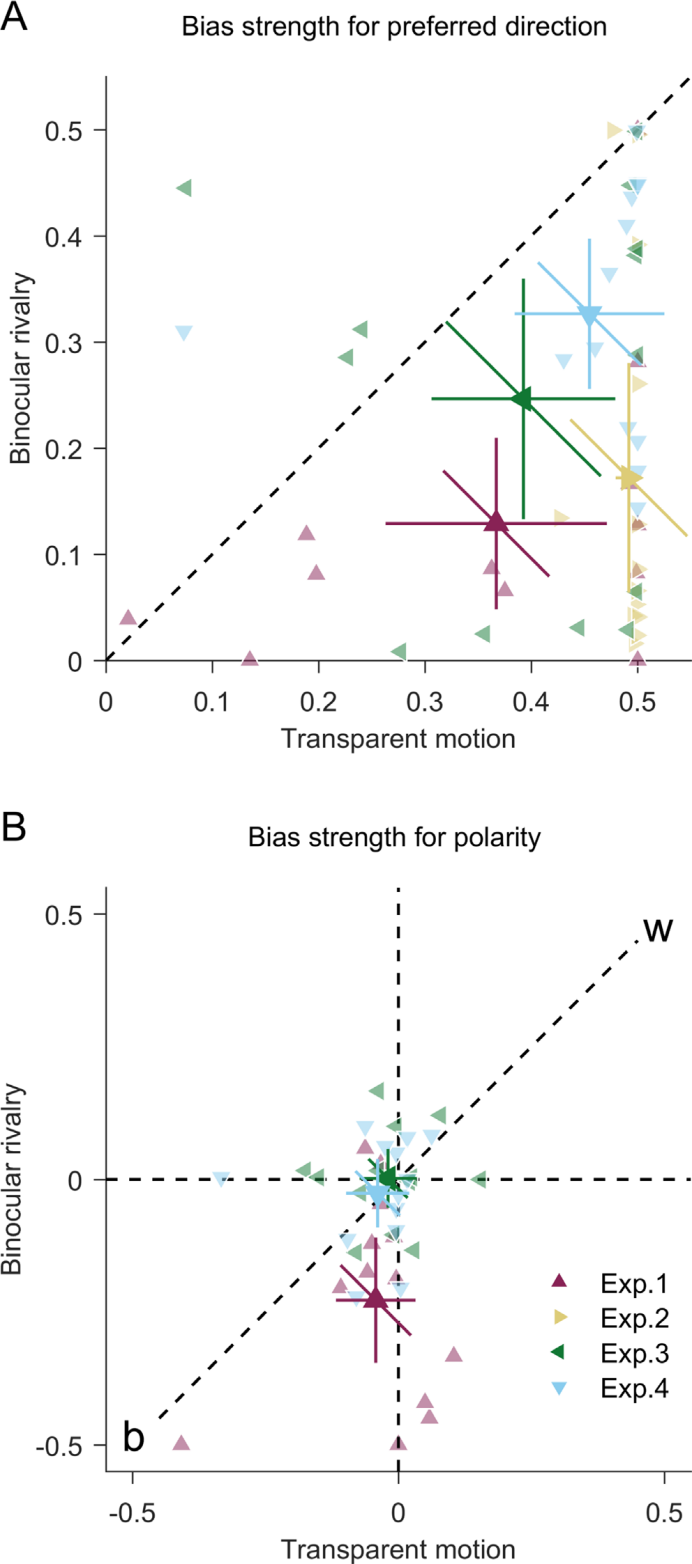
(A) Direction bias strength in transparent motion and binocular rivalry conditions. (B) Polarity bias strength in transparent motion and binocular rivalry conditions. Negative values indicate biases toward black polarity (‘b’) and positive values indicate biases toward white polarity (‘w’). Experiment 2 does not contain polarity as a feature, and therefore is not plotted for polarity biases. (A and B) Large symbols indicate the average across participants; small symbols indicate data of individual participants. Horizontal and vertical error bars indicate 95% between-subject confidence intervals. The diagonal error bars indicate 95% confidence intervals of the within-subjects difference between the *x* and *y* values and need to be compared with the dashed diagonal representing equal *x* and *y* values.

To compare the bias strength, we performed a condition (transparent motion, binocular rivalry) × bias (direction bias, polarity bias) repeated measures analysis of variance (ANOVA). The two-way interaction between condition and bias was significant, *F*(1, 12) = 31.149, *p* < 0.001, *BF*_10_ = 878.217 (Bayes-factor analysis; for a review, see Jarosz & Wiley, 2014). In transparent motion, the direction bias (*M* = 0.367, *SD* = 0.172) was stronger than the polarity bias (*M* = 0.075, *SD* = 0.106) (Figure 2A), *t*(12) = 4.273, *p* = 0.001, *BF*_10_ = 37.06. The direction bias was stronger in transparent motion than in binocular rivalry (*M* = 0.129, *SD* = 0.134) (Figure 3A), *t*(12) = 5.205, *p* < 0.001, *BF*_10_ = 145.3. The polarity bias was stronger in binocular rivalry (*M* = 0.241, *SD* = 0.177) than in transparent motion, *t*(12) = 3.679, *p* = 0.003, *BF*_10_ = 15.03, and, in general, black dots were preferred over white dots (Figure 3B). In binocular rivalry, there were no significant differences between direction and polarity biases, *t*(12) = 1.399, *p* = 0.187, *BF*_10_ = 0.617, and direction and eye-of-origin biases, *t*(12) = 0.826, *p* = 0.425, *BF*_10_ = 0.372, and polarity and eye-of-origin biases, *t*(12) = 2.057, *p* = 0.062, *BF*_10_ = 1.368.

### Experiment 2: Eliminated polarity bias

Because the polarity bias was stronger for binocular rivalry than for transparent motion in Experiment 1, it might be that the strong polarity bias in binocular rivalry limited the potential motion direction bias. To eliminate the influence of the polarity bias in Experiment 2, we used the same polarity for both dot clouds and participants had to report the motion direction they perceived in front (in the transparent motion condition) or the motion direction they perceived dominant (in the binocular rivalry condition).

Even when we eliminated the polarity bias, the direction bias was still stronger in transparent motion (*M* = 0.492, *SD* = 0.021) than in binocular rivalry (*M* = 0.172, *SD* = 0.179) (Figure 3A), *t*(12) = 6.34, *p* < 0.001, *BF*_10_ = 685.3.

Instead, the eye-of-origin bias became stronger in binocular rivalry (Figure 2B): it was stronger in Experiment 2 (*M* = 0.305, *SD* = 0.182) than in Experiment 1 (*M* = 0.093, *SD* = 0.116), *t*(24) = 3.538, *p* = 0.002, *BF*_10_ = 20.29.

### Experiment 3: Calibrated polarity bias

By eliminating the polarity biases in Experiment 2, the eye-of-origin instead of the motion direction bias got stronger in binocular rivalry. In Experiment 3, while using two different polarities for the two dot clouds to facilitate perceptual dominance in the binocular rivalry condition, we tried to minimize the polarity bias for each participant by calibrating the relative contrast of white and black dots relative to the gray background.

We performed a condition (transparent motion, binocular rivalry) × bias (direction bias, polarity bias) repeated measures ANOVA. The two-way interaction between condition and bias was significant, *F*(1, 24) = 9.774, *p* = 0.005, *BF*_10_ = 8.788. In transparent motion, the direction bias (*M* = 0.393, *SD* = 0.143) was stronger than the polarity bias (*M* = 0.067, *SD* = 0.058) (Figure 2A), *t*(12) = 6.269, *p* < 0.001, *BF*_10_ = 624.5. In binocular rivalry, the direction bias (*M* = 0.246, *SD* = 0.187) and the eye-of-origin bias (*M* = 0.253, *SD* = 0.188) were stronger than the polarity bias (*M* = 0.064, *SD* = 0.063), *t*(12) = 3.685, *p* = 0.003, *BF*_10_ = 15.16; *t*(12) = 3.082, *p* = 0.01, *BF*_10_ = 6.037, respectively. The direction bias was stronger in transparent motion than in binocular rivalry (Figure 3A), *t*(12) = 2.187, *p* = 0.049, *BF*_10_ = 1.633.

In the further analysis, we compared the polarity bias strength between Experiments 1 and 3 to check whether the polarity bias calibration in Experiment 3 was effective. Indeed, the polarity bias was weaker in Experiment 3 than in Experiment 1, *t*(24) = 3.396, *p* = 0.002, *BF*_10_ = 15.377. Moreover, we compared the eye-of-origin bias strength between Experiments 1 and 3 to check whether the polarity bias calibration in Experiment 3 affected the eye-of-origin bias. Interestingly, the eye-of-origin bias was stronger in Experiment 3 than in Experiment 1, *t*(24) = 2.606, *p* = 0.016, *BF*_10_ = 3.718. Hence, the calibration of polarity bias lead to an increase of the eye-of-origin bias, just like the absence of the polarity bias in Experiment 2.

### Experiment 4: Calibrated polarity bias and eliminated eye-of-origin bias

In Experiments 2 and 3, the direction bias in binocular rivalry was still weak even when the polarity bias was minimized by matching the contrasts or eliminated by using identical polarities for both dot clouds. However, in those experiments, the eye-of-origin bias was quite pronounced. Therefore we performed Experiment 4 to minimize the biases for polarity and eye-of-origin at the same time. In a previous binocular rivalry study (Kovács, Papathomas, Yang, & Fehér, 1996), the coherency of conventional stimuli was broken and replaced by complementary patches of intermingled rivalrous images. In this study, pattern coherency could drive perceptual alternations, and the patches were reassembled into coherent forms by most observers. Based on this finding, we spilt each of the two dot clouds into two parts and showed them to different eyes. If participants could reassemble these split parts of the dot clouds into one coherently moving layer, we could efficiently minimize the eye-of-origin effect. In addition, we performed the polarity contrast calibration as in Experiment 3 to minimize the polarity bias.

We performed a condition (transparent motion, binocular rivalry) × bias (direction bias, polarity bias) repeated measures ANOVA. As a result, the two-way interaction between condition and bias was significant, *F*(1, 24) = 15.329, *p* = 0.001, *BF*_10_ = 664.99. In transparent motion, the direction bias (*M* = 0.455, *SD* = 0.116) was stronger than the polarity bias (*M* = 0.054, *SD* = 0.090) (Figure 2A), *t*(12) = 7.120, *p* < 0.001, *BF*_10_ = 1838.5. In binocular rivalry, the direction bias (*M* = 0.327, *SD* = 0.117) was stronger than the polarity bias (*M* = 0.084, *SD* = 0.067), *t*(12) = 5.753, *p* < 0.001, *BF*_10_ = 312.4, and also compared with the direction bias in Experiment 1 and 2 within same condition (Figure 2B), *t*(24) = 4.011, *p* < 0.001, *BF*_10_ = 52.73; *t*(24) = 2.605, *p* = 0.016, *BF*_10_ = 3.716, respectively. However, the direction bias was still stronger in transparent motion than in binocular rivalry (Figure 3A), *t*(12) = 2.883, *p* = 0.014, *BF*_10_ = 4.469.

### Comparison of directional biases in transparent motion and binocular rivalry

**Figure 4.**
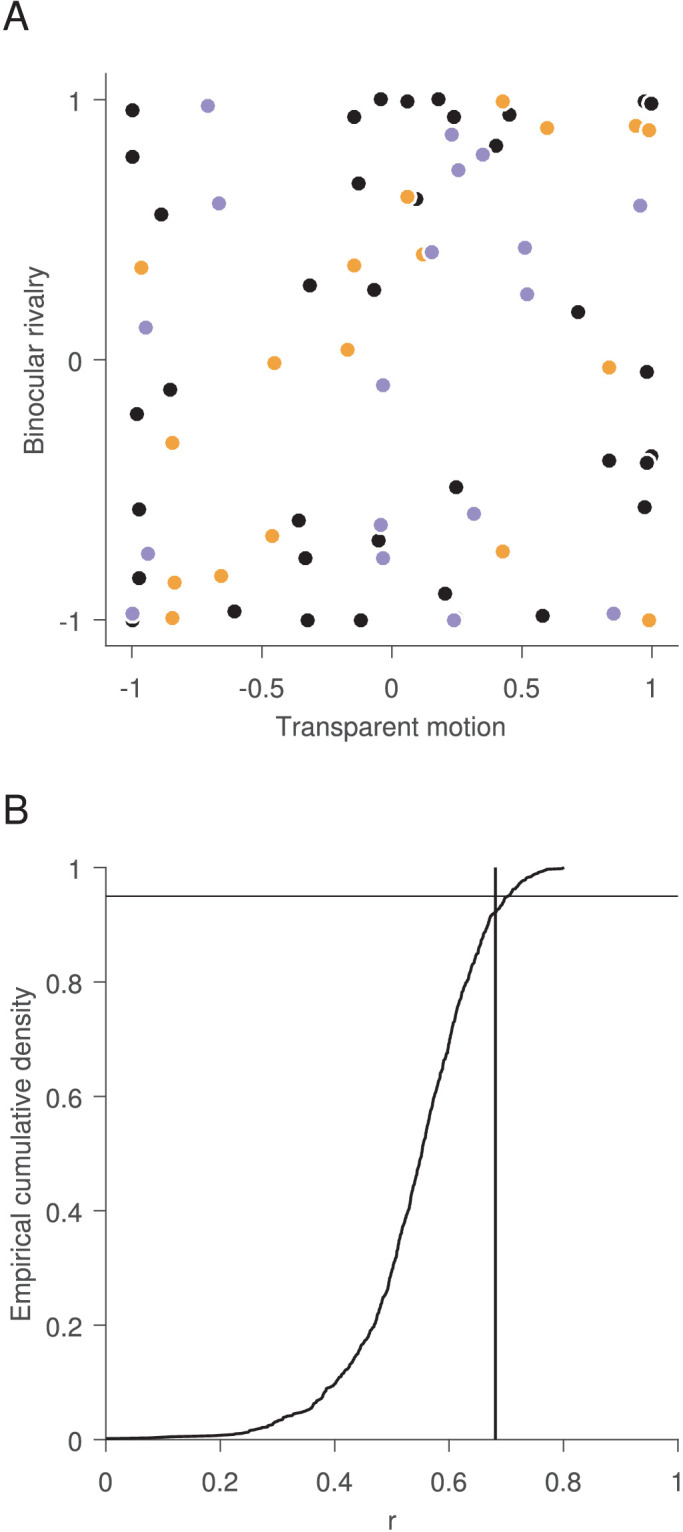
Correlation between preferences in transparent motion and binocular rivalry for all experiments. (A) Circular correlation between preferred directions (Equation 3). The *x* and *y* values represent the sine of the difference between the preferred directions in the respective condition and their mean. The regression lines indicate the circular correlation; circles indicate individual data points. The different colors indicate the whole sample (black), data below (orange) or above (violet) the median bias strength in binocular rivalry. (B) Correlation between preferred axes. Preferences in binocular rivalry are normalized such that the preference axis is preserved while the direction is constrained to point into the same half circle as the preference in transparent motion. The thick black line shows the distribution of correlation coefficients with random pairing of normalized preferences in transparent motion and binocular rivalry. The vertical line indicates the correlation coefficient with the actual pairing of 0.681. The horizontal line indicates the critical value of 95%.

Ultimately, we wanted to compare the preferred directions in transparent motion and binocular rivalry in all four experiments. For the following analyses, we first excluded observers with extremely weak directional preferences in binocular rivalry of less than 0.04, which essentially represents the absence of a directional preference (participants #101, 90, 87, 128, 130, 135, 137, 139 and 144; Figure A1). In addition, we had five observers that took part in more than one experiment. To avoid the issue of pseudoreplication (Lazic, 2010), we selected only the session with the strongest direction bias in binocular rivalry for those observers. This left us with a sample of 37 pairs of biases in transparent motion and binocular rivalry. As in a previous study (Schütz, 2014), we calculated a circular correlation coefficient between the preferred directions in transparent motion and binocular rivalry (Figure 4A). This correlation was neither significant for the whole sample (*r*(35) = 0.120, *p* = 0.437) nor for samples below (*r*(16) = 0.440, *p* = 0.090) or above (*r*(16) = 0.120, *p* = 0.625) the median bias strength in binocular rivalry. This suggests that there was no evidence for a simple and direct relationship between the preferred directions in transparent motion and binocular rivalry.

In the next step, we tested if there might be a more complex relationship between the directional biases. If the motion direction perceived in front is more salient than the one in the back in transparent motion (Chopin & Mamassian, 2011), one might assume that the motion direction seen in *front* should coincide with the dominant motion direction in binocular rivalry. Nevertheless, it also might be that the motion direction seen in the *back* is more salient (Schütz, 2011, 2012) and coincides with the dominant motion direction in binocular rivalry. If this pairing varies between observers, only the axes, but not the direction of preferences could be correlated across observers. We therefore normalized the preferred directions in binocular rivalry, such that the axis of preference is preserved while the direction on this axis is constrained to be aligned with the preferred direction in transparent motion, such that both preferences point toward the same half circle. The resulting circular correlation coefficient (*r*(35) = 0.681) was exceeded by 7.90% of correlation coefficients based on 1000 datasets in which preferred directions in transparent motion and binocular rivalry were randomly reshuffled (Figure 4B). This suggests that preferences were not significantly correlated between transparent motion and binocular rivalry, even when only preference axes were considered.

## Discussion

Four experiments were conducted to assess and compare idiosyncratic perceptual biases in transparent motion and binocular rivalry. In Experiment 1, we presented two dot clouds that were moving in opposite directions and that had opposite contrast polarity (black vs. white). We found a double dissociation, with strong direction biases in transparent motion but strong contrast polarity biases in binocular rivalry. In Experiment 2, we used the same contrast polarity for the two dot clouds to remove the polarity biases found in binocular rivalry in Experiment 1. This led to an increase in the eye-of-origin biases in binocular rivalry but left the direction biases largely unaffected. In Experiment 3, we calibrated the polarity contrast to minimize the polarity biases during binocular rivalry. The polarity biases vanished when the polarity contrast was calibrated, but the eye-of-origin biases were still present during binocular rivalry. In Experiment 4, we split the motion dot clouds into two parts and showed each part to different eyes to minimize the biases for polarity and eye-of-origin at the same time in binocular rivalry. The polarity contrast was calibrated as in Experiment 3. As a result, we found stronger direction biases in binocular rivalry compared with the other experiments. However, in general, the direction biases in binocular rivalry were weaker than in transparent motion across all experiments. Moreover, the directional preferences showed no significant circular correlation between transparent motion and binocular rivalry.

Replicating previous findings in transparent motion (Mamassian & Wallace, 2010; Schütz, 2014; Wexler, Duyck, & Mamassian, 2015), we observed strong idiosyncratic preferences for motion direction but rather weak preferences for contrast polarity. The results were reversed in binocular rivalry, showing weak preferences for motion direction but strong preferences for contrast polarity. These findings indicate that idiosyncratic preferences in a visual feature (motion direction) can be dissociated at different stages of visual information processing. The weak directional preferences in binocular rivalry are in stark contrast to findings from other types of ambiguous stimuli (Schütz, 2014; Wexler, Duyck, & Mamassian, 2015; Wexler, 2018), suggesting that motion direction plays a less important role in binocular rivalry.

Andersen and Bradley (1998) proposed that V1 and MT play an important role in the processing of structure-from-motion. Structure-from-motion (Wallach & O'Connell, 1953) is the perception of depth induced by retinal motion similar as in transparent motion. Andersen and Bradley (1998) argued that there are two serial motion processing stages from V1 to MT/MST (the medial superior temporal area). In the first stage, motion measurements are made in area V1. The next stage occurs in MT, where direction opponency suppresses noise as part of a surface reconstruction process. Supporting this idea, Snowden and colleagues (1991) found that direction-selective V1 neurons generally give the same response to a stimulus moving in their preferred direction, whether or not a second stimulus is present and moving in the opposite direction. However, there was strong suppression of MT neurons under these transparent conditions. Qian and Andersen (1994) showed that the perception of transparent motion can be eliminated if the dots moving in opposite directions are all locally paired with each other. Interestingly, direction selective neurons in V1 could not reliably distinguish between the locally paired and unpaired motion, whereas neurons in MT were more activated by the unpaired motion. Qian and Andersen also found opponent-direction suppression in MT cells and argued that their findings are consistent with the two-stage model. Moreover, Bradley, Qian, and Andersen (1995) showed that inhibition in area MT occurs mainly between motion signals with similar disparities. Based on these findings, Andersen and Bradley (1998) suggested inhibitory interactions between MT cells tuned for opposite directions and similar depths and excitatory connections between neurons tuned for opposite directions and different depths. These arguments agree with the findings from Krug and colleagues (2013). Krug and colleagues found that electrical microstimulation of neuronal populations with conjoint tuning for motion and depth in V5/MT can bias the perceived rotation direction of structure-from-motion stimuli. This suggests a causal role of the conjoint representation of motion and depth signals.

However, MT is not the only relevant area in this field. Previous studies suggested that the early visual pathway might play an important role for interpreting transparent motion and also binocular rivalry. Schütz and Mamassian (2016) found that idiosyncratic directional preferences appear to be a fast process that relies on early 1D motion signals. This finding suggests that the directional bias in transparent motion could only originate in V1 or in the early MT response because the actual 2D motion signal is resolved afterward (Pack & Born, 2001; Pack, Livingstone, Duffy, & Born, 2003). Hence the finding by Schütz and Mamassian (2016) puts an upper bound on the neuronal origin of directional biases in transparent motion. We believe that the dissociation of biases between binocular rivalry and transparent motion and the overall weak biases in binocular rivalry in our present results puts a complementary, lower bound on the neural origin of directional biases. Previous studies showed that binocular rivalry depends on the reciprocal inhibition between monocular neurons early in the visual processing stream (Blake, 1989; Baker & Graf, 2009; Klink, Brascamp, Blake, & van Wezel, 2010; Brascamp, Sohn, Lee, & Blake, 2013). Therefore we speculate that the directional biases in transparent motion could originate at two different stages in processing. The first possibility is that while the eye-based binocular rivalry seems to be resolved in the early visual stream (i.e., V1; Tong, Meng, & Blake, 2006), the directional bias in transparent motion might rely on early MT responses rather than V1 responses. The second possibility is that directional biases in transparent motion and binocular rivalry might depend on different subpopulations in V1. In this case, we suggest that, in lower visual pathways (e.g., V1), the directional bias in transparent motion might rely on the neural subpopulation that processes binocular information, whereas the directional biases in binocular rivalry might rely on the other subpopulation that processes monocular information. Therefore both monocular and binocular neurons in the lower visual pathway might contribute to the interpretation of the ambiguous stimuli, but each neural population is recruited for different classes of ambiguous stimuli. In addition, for the stimulus-driven binocular rivalry (Diaz-Caneja, 1928; translated by Alais, O'Shea, Mesana-Alais, & Wilson, 2000; Kovács, Papathomas, Yang, & Fehér, 1996; Logothetis, Leopold, & Sheinberg, 1996), which we used in Experiment 4, the interactions between binocular neurons across V1 to V4+ (especially in V4+) seems to play a critical role for resolving the rivalry (Leopold & Logothetis, 1996). Even though our present results narrowed down the possible neuronal origins of directional biases in transparent motion to some degree, further studies would be necessary to disentangle the possibilities we discussed.

Recent studies showed no (Brascamp, Becker, & Hambrick, 2018; Cao, Wang, Sun, Engel, & He, 2018) or only a weak (Steinwurzel, Animali, Cicchini, Morrone, & Binda, 2020) correlation in percept durations between binocular rivalry and structure-from-motion. This might imply that there are two independent rather than one shared control mechanism for inferences in binocular rivalry and structure-from-motion. Although these findings emphasize the temporal dissimilarity between the two phenomena, our present results emphasize the spatial dissimilarity by showing differences in directional biases between the phenomena.

In binocular rivalry, we measured biases in preferred motion direction, contrast polarity, and eye-of-origin. By applying different experimental settings, we tried to reduce the biases other than the directional bias. However, altough we succeeded to reduce or minimize the biases other than the directional bias, it seems that there might be a hierarchical priority of visual information for the solution of binocular rivalry. Based on our findings, polarity biases have precedence over eye-of-origin biases and eye-of-origin biases have precedence over directional bias. This might tell us the priorities of solving various rivalrous information during binocular rivalry. In addition, our results showed a strong bias for dominance of black over white in binocular onset rivalry. Several previous studies reported this black-white asymmetry and found that responses to light decrements are usually faster or stronger than responses to light increments (see Lu & Sperling, 2012, for a review). In early vision, there are two pathways processing contrast polarity information: the ON pathway responding to light increments and the OFF pathway responding to light decrements (Schiller, 1995). Differences between the ON and OFF pathways have been reported in early vision even from the retina to V1 (He & MacLeod, 1998; Jin et al., 2008; Balasubramanian & Sterling, 2009; Yeh, Xing, & Shapley, 2009; Xing, Yeh, & Shapley, 2010; Jin, Wang, Lashgari, Swadlow, & Alonso, 2011; Komban, Alonso, & Zaidi, 2011; Komban et al., 2014). Moreover, a recent study reported significantly lower detection thresholds when using black motion dots as opposed to white motion dots in pigeons (Nankoo, Madan, Spetch, & Wylie, 2017). In our study, especially in the binocular rivalry condition of Experiment 1, the strong polarity bias might have occurred because black and white contrast polarities were presented into different eyes and the black RDKs could lead to faster and stronger neuronal activiation than the white RDKs. This might explain the precedence of polarity biases over other types of biases in binocular rivalry of Experiment 1. We speculate that presenting strong black and white contrast polarities into each eye might amplify the differences between the ON and OFF pathways and cause strong polarity biases in binocular rivalry, whereas presenting both polarities to the same eye might cause mild perceptual biases. Further investigation would be needed to find out more about the neural basis and the hierarchy of perceptual biases.

## Conclusions

Our study showed that idiosyncratic directional preferences were generally stronger in transparent motion compared with binocular rivalry. Moreover, the directional preferences did not correlate between transparent motion and binocular rivalry. Therefore we argue that idiosyncratic directional preferences in transparent motion and binocular rivalry originate at different processing stages of motion perception. Moreover, the strength of perceptual biases showed a striking double-dissociation: binocular rivalry was dominated by biases in contrast polarity and transparent motion was dominated by biases in motion direction.
